# Rectal artemisinins for malaria: a review of efficacy and safety from individual patient data in clinical studies

**DOI:** 10.1186/1471-2334-8-39

**Published:** 2008-03-28

**Authors:** Melba Gomes, Isabela Ribeiro, Marian Warsame, Harin Karunajeewa, Max Petzold

**Affiliations:** 1 UNICEF/UNDP/World Bank/WHO Special Programme for Research and Training in Tropical Diseases, World Health Organization, 20 Avenue Appia, Geneva 27, Switzerland; 2 Division of International Health (IHCAR), Karolinska Institutet, Stockholm, Sweden; 3 Rectal Artesunate Study Team, National Institute for Medical Research (NIMR), Dar es Salaam, Tanzania; 4 University of Western Australia, Crawley, Western Australia and Papua New Guinea Institute of Medical Research, Goroka EHP 441, Papua New Guinea; 5 Nordic School of Public Health, Göteborg, Sweden; 6 Global Malaria Programme, World Health Organization, 20 Avenue Appia, Geneva 27, Switzerland; 7 Drugs for Neglected Diseases – DNDi, Regional Office – Latin America, Rua Santa Luzia 651/11° andar, 20030-041 – Rio de Janeiro, Brasil

## Abstract

**Background:**

Rectal administration of artemisinin derivatives has potential for early treatment for severe malaria in remote settings where injectable antimalarial therapy may not be feasible. Preparations available include artesunate, artemisinin, artemether and dihydroartemisinin. However each may have different pharmacokinetic properties and more information is needed to determine optimal dose and comparative efficacy with each another and with conventional parenteral treatments for severe malaria.

**Methods:**

Individual patient data from 1167 patients in 15 clinical trials of rectal artemisinin derivative therapy (artesunate, artemisinin and artemether) were pooled in order to compare the rapidity of clearance of *Plasmodium falciparum *parasitaemia and the incidence of reported adverse events with each treatment. Data from patients who received comparator treatment (parenteral artemisinin derivative or quinine) were also included. Primary endpoints included percentage reductions in parasitaemia at 12 and 24 hours. A parasite reduction of >90% at 24 hours was defined as parasitological success.

**Results:**

Artemisinin and artesunate treatment cleared parasites more rapidly than parenteral quinine during the first 24 hours of treatment. A single higher dose of rectal artesunate treatment was five times more likely to achieve >90% parasite reductions at 24 hours than were multiple lower doses of rectal artesunate, or a single lower dose administration of rectal artemether.

**Conclusion:**

Artemisinin and artesunate suppositories rapidly eliminate parasites and appear to be safe. There are less data on artemether and dihydroartemisinin suppositories. The more rapid parasite clearance of single high-dose regimens suggests that achieving immediate high drug concentrations may be the optimal strategy.

## Background

In 1985, the Journal of Traditional Chinese Medicine described the satisfactory efficacy of *qinghaosu *suppositories in 100 patients with *P. falciparum *malaria, 4 of whom had cerebral malaria [1]. *Qinghaosu *(artemisinin) derivatives were soon recognised as having powerful antimalarial activity [2] and a variety of formulations have since been developed[3]. The efficacy and safety of oral and parenteral artemisinin derivatives have been widely studied for both uncomplicated and severe malaria [4-7] and these drugs form the basis of current antimalarial treatment policy in most countries in the world [8].

During the past 10 years, the WHO has directed the development of the highly active artemisinin derivative, artesunate, to assess its value in settings where it might be given rectally as a substitute for injectable treatment. The rationale for the development was that, without effective treatment, *P. falciparum *malaria can progress to severe malaria and death within a matter of hours. The artemisinin derivatives have been shown to have potent activity against early trophozoite forms and to rapidly reduce heavy parasite infections[3]. Therefore it was postulated that when given as a suppository in areas where patients cannot immediately access injectable therapy, they might confer therapeutic advantages in preventing parasite development to the more pathological stages that cause organ complications in severe malaria [9]. Rectal preparations have the advantage of being easy to administer in rural areas; therefore it is anticipated that rectal administration of an artemisinin derivative in remote settings might "buy time" by halting or slowing the progress of disease while a patient is being transported to a health-care facility equipped to provide definitive treatment. Their utility would consequently be greatest in areas where access to injectable therapy is poor or does not exist. The clinical evidence accumulated in the initial phase of this development focused on measures of parasite reduction – a well-established indicator of clinical effect in the evaluation of antimalarial drugs.

In addition to artesunate, other artemisinin derivatives formulated for rectal administration now include artemisinin, dihydroartemisinin and artemether. The available published results suggest that all achieve a rapid, consistent clinical response in regions where studies have been undertaken, despite considerable inter-individual pharmacokinetic variability[10-14]. However the different artemisinin derivatives have different physicochemical properties in adminstration; consequently some preparations might be more rapidly absorbed than others [11,13,15-18]. Most clinical trials have employed small sample sizes and none have directly compared the efficacy of the different rectally administered artemisinin derivatives with one another. In addition, substantial heterogeneity exists with respect to the dosing schedules employed. There is therefore considerable uncertainty regarding the optimal preparation and dosing schedules to use.

Given the different aims, design, location, patient demographics and disease severity of clinical trials, we review individual patient data from several trials to establish whether there are significant differences in efficacy and safety of different artemisinin-based suppositories in the first 24 hours post treatment. More specifically we evaluate: i) the efficacy of rectal artemisinins in comparison with conventional treatments for severe malaria (including parenteral quinine and parenteral artemisinins); ii) the comparative efficacy of different artemisinin derivatives for rectal use (namely, artesunate vs artemisinin vs artemether); iii) the efficacy of different dosing regimens (single vs multiple dosing) and total dose administered; iv) the overall safety profile of rectally administered artemisinins and differences between different drugs and regimens.

## Methods

### Search strategy and selection criteria

Electronic searches of the National Library of Medicine's MEDLINE database, Current Contents database and manual searches of selected specialty journals were performed to identify all the pertinent literature. MEDLINE database engines (Ovid, PubMed and GratefulMed) were used with the keywords "rectal", "artemisinin", and "treatment". The search was further refined with the words "artesunate", "artemether", "artemisinin", "dihydroartemisinin", "suppositories", and "rectocaps" from 1980 to March 2006. Reference lists from qualitative topic reviews and published clinical trials in English were also searched. We attempted to obtain the original individual patient data from all studies, regardless of publication status.

Selection criteria for inclusion of studies were clinical trials that assessed the efficacy of a rectal artemisinin-based preparation where individual patient data (inclusion/exclusion criteria, age, parasitological status at baseline and parasitology post treatment) were available. Comparative and non-comparative studies were included, regardless of study design, geographical area, patient age group, disease severity or the artemisinin derivative used. Safety data and information on dosage was specifically requested from the individual study investigators if this was not provided or evident from a publication. Data extraction was conducted by two investigators (MP, IR) for efficacy data and two investigators (MW, MG) for safety.

### Efficacy endpoints

The main focus of the review was on the assessment of parasite reduction and clinical response of patients during the first 24 hours following treatment. This was considered most appropriate, given the intended indication as emergency pre-referral treatment, where ameliorating disease progression within 24 hours, i.e. while the patient is being transported to a clinic or hospital, is likely to be most important.

All efficacy definitions used are consistent with the scientific literature. Parasite reduction ratio (PRR) at 12 and 24 hours was assessed as the percent reduction of parasitaemia at 12 and 24 hours from baseline parasitaemia. Parasitological success was defined as the absence of clinical deterioration from baseline and a PRR at 24 hours of ≥ 90%. In the analysis (see further below) both the continuous variable PRR and the binary variable of ≥ 90% parasite reduction were used, the former to define efficacy at 12 hours, and the latter to define efficacy at 24 hours.

Because definitions of parasite and fever clearance times differed from study to study, these time-to-event variables were re-derived from the serial parasite density estimations and temperatures for individual patients. Parasite clearance and fever clearance time were defined as the time at which the first negative blood smear or normal temperature (<37.5°C axillary or <38.0°C oral) was recorded. The effect of consolidation treatment on recrudescence of the infection during follow-up was evaluated from baseline to the reappearance of parasitaemia.

### Safety endpoints

Except in two trials, laboratory markers of safety (including haematological and biochemical indices) were not available. Safety analyses were consequently restricted to clinical descriptions of reported adverse events. In the absence of prospective standardised methods for defining, assessing, reporting and classifying adverse events across all trials, and due to inherent difficulties in clinically distinguishing drug side-effects from manifestations of malarial infection, principal investigators who contributed data were asked to re-review individual patient data retrospectively and reassess all reported adverse events. Ideally this was performed directly from case record forms where archived data were accessible. Each reported event was thus re-classified by the clinical investigator as being either "unlikely", "possibly", "probably" or "definitely" due to the treatment. Those events considered possibly, probably or definitely drug-related were thereafter re-classified as "potentially drug-related" for the purposes of the pooled analysis.

### Statistical analysis

Original data from different studies were merged into a master data set. In the analysis mainly hierarchical models were used, with treatment arm as the second level of clustering. In some cases within-study comparisons of different treatment arms was possible and methods for meta-analysis were then accordingly applied in the analysis of PRR. A hierarchical mixed model applying the DerSimonian & Laird method was used with the estimate of heterogeneity being taken from the inverse variance fixed-effect model [19].

Most of the analyses, however, were based on comparisons of treatment arms from different studies, here called pooled analyses, and conducted as follows: For PRR, linear mixed effect models with random intercepts were applied. In the analysis at 12 hours, an identity link (normal distribution) was used. At 24 hours a large proportion of the PRR values for the artemisinins were close to 100% at 24 hours, and these were then categorised into binary observations (PRR >90% versus <90%). A logistic link was then used in the analysis. It should be noted that crude mean values of PRR at 24 hours using the identity link are also given for description. We systematically examined the effect (stepwise backward elimination of covariates not reaching a significance level of p = 0.05) of the following covariates within all analyses: baseline parasitaemia, age, region, total treatment dose provided within the initial 12 and 24 hours, and severity of disease as defined by the evaluating clinician.

Time-to-event analyses, including time to parasite and fever clearance and the efficacy of the consolidated treatment in suppressing parasitaemia post treatment (reappearance of parasites), were represented using crude Kaplan-Meier plots (these are provided as graphs only where the difference in estimates was significant). Hazard-ratios were estimated using a non-hierarchical Cox regression model applying the same stepwise backward elimination of covariates as above. Intra-rectal treatments were compared to parenteral treatment when followed by the same consolidation treatment in one analysis and intra-rectal treatments followed by different consolidation treatments were compared in a second analysis. Time to parasite clearance, age, region, disease severity, parasitaemia at 72 hours (often the start of consolidation treatment) were assessed as covariates through stepwise backward elimination of covariates.

All statistical computations were performed with Stata for Windows (version 8 and 9.2, Stata Corporation, College Station, Texas-USA).

## Results

### Studies included

A total of 27 studies were identified in patients using the search criteria; 4 studies were in healthy normal volunteers [11,15,16,18]. Twelve studies were not included in the analysis: 5 trials were on pharmacokinetics – 8 to 15 patients per trial, total of 59 patients [14,17,20-22] with no efficacy data available. This left 7 trials, 242 patients with uncomplicated malaria[23-26] and 185 patients with severe and complicated malaria treated with rectal artemisinins[23,27-29] that were not available to be included in our analyses.

Individual patient data were available from 15 clinical trials (Table 1). There were 4 randomised, controlled, clinical trials with parenteral quinine as comparator: Molyneux1997–8 [30], Barnes1998 [30], Phuong1992–5 [31], Aceng2002–3 [32], and 6 trials in which the comparator was another artemisinin given parenterally or orally in moderately severe, hyperparasitaemic and severe patients: Krishna1996 [10], Looareesuwan1996 (unpublished), VanVugt1997–9 (unpublished), Vinh 1997–9 [33], Hien1998 (unpublished), Karunajeewa2003–4 [34]. The remaining trials used a different dosage or treatment regimen with the same artemisinin-based rectal preparation as a comparator: Looareesuwan1995 [35], Looareesuwan2000 (unpublished), Than1998 (unpublished). There were 2 non- comparative trials – Bhatt1994–5 [36], Karunajeewa2001 [37]. We were provided with an additional 10 individual patient observations not included in one published trial (Phuong1992–5) [31] which completed with fewer patients than planned due to recruitment difficulties, but where the team continued to collect clinical descriptions of severe malaria in children, using rectal artemisinins which the hospital preferred to quinine. The data from these 10 cases were added to the pooled analyses.

**Table 1:** Studies for which individual patient data was provided, by study, treatment and number of patients

**Region**	**Country**	Study Identification* [Ref]	**Treatment Group**	**Number of Patients Studied**			**Total Number of Patients**
					
				Moderately Severe	Severe	Uncomplicated	
**Africa**	Ghana	Krishna 1996 [10]	Artesunate ir, single dose	23			23
			Artemisinins** parenteral	11			11
	Kenya	Bhatt 1994–5 [36]	Artesunate ir, multiple dose		23		23
	Malawi	Molyneux 1997–8 [30]	Artesunate ir, single dose	86			86
			Quinine parenteral	22			22
	South Africa	Barnes 1998 [30]	Artesunate ir, single dose	27			27
			Quinine parenteral	8	6		14
			Quinine parenteral+ Artesunate ir		5		5
	Uganda	Aceng 2002–3 [32]	Artemether ir, single dose		51		51
			Quinine parenteral		52		52

**Total Number of Patients – Africa**				**177**	**137**		**314**

**Asia- Oceania**	Myanmar	Than 1998	Artesunate ir, multiple dose		100		100
	Papua New Guinea	Karunajeewa 2001 [37]	Artesunate ir, multiple dose			48	48
	Papua New Guinea	Karunajeewa 2003–4 [34]	Artesunate ir, multiple dose		41		41
			Artemisinins parenteral		38		38
	Thailand	Looareesuwan 1996	Artesunate ir, single dose	26			26
			Artemisinins parenteral	24			24
	Thailand	Van Vugt 1997–9	Artesunate ir, single dose	44			44
			Artesunate po	17			17
	Thailand	Looareesuwan 1995 [35]	Artesunate ir, multiple dose	60			60
	Thailand	Looareesuwan 2000	Artesunate ir, single dose	69			69
	Vietnam	Phuong 1992–5 [31]	Artemisinin, multiple dose		46		46
			Artemisinins parenteral		40		40
			Quinine parenteral		35		35
	Vietnam	Vinh 1992–4 [33]	Artemisinin ir, multiple dose		52		52
			Artemisinins parenteral		123		123
	Vietnam	Hien 1998	Artemisinin ir, single dose		46		46
			Artesunate ir, single dose		44		44

**Total Number of Patients – Asia**				**240**	**565**	**48**	**853**

**Grand Total**				**417**	**702**	**48**	**1167**

* Study Identification: Investigator name and year of patient enrolment [References are included where available].

** Artemisinins parenteral: this consisted of Artemether or Artesunate administered via intra-muscular or intravenous route

Altogether the studies included in this analysis enrolled a total of 1167 patients in 37 separate treatment arms. Five patients who simultaneously received rectal artesunate and quinine were excluded, leaving a total of 1162 patients included in the analysis. Of these 786 had been treated with rectal administration of an artemisinin derivative and 376 with a comparator drug which was either a parenteral artemisinin (236) oral artesunate (17) or parenteral quinine (123). The majority of included patients treated with a rectal artemisinin were from mainland South East Asia (487) or Papua New Guinea (89), with relatively fewer patients from Africa (210). Thirty-one percent of patients were children under 5, 11.8% children aged 6–10, 21.3% adolescents aged 11–20 years and 36% adults over 20 years of age. Treatment exposure information is provided in Table 2.

**Table 2:** Summary of Age and Doses used in clinical trials by type of therapy(mg/kg)

Treatment group	Median age, years (range)	Dose(mg/kg) at Initiation of therapy (mean ± SD)	Total dose over first 12 hours (mg/kg) (mean ± SD)	Total dose over first 24 hours (mg/kg) (mean ± SD)
Artemether ir*, single dose	2.08 (0.42 – 5)	6.7 ± 1.19	6.7 ± 1.19	6.7 ± 1.19
Artemisinin ir, single dose	19 (4 – 41)	20.0 ± 0	20.0 ± 0	20.0 ± 0
Artemisinin ir, multiple dose	20 (0.7 – 62)	31.5 ± 8	45.1 ± 14	45.1 ± 14
Artesunate ir, multiple dose	19 (1.3 – 80)	6.65 ± 4	8.1 ± 4.17	14.8 ± 7.64
Artesunate ir, single dose – 10 mg/kg	12 (1.33 – 58)	9.4 ± 2.47	9.4 ± 2.47	9.4 ± 2.53
Artesunate per os	6 (0.92 – 15)	4.0 ± 0	4.0 ± 0	4.0 ± 0
Artemisinins parenteral	18 (0.5 – 66)	2.94 ± 0.61	2.94 ± .0.6	3.43 ± 0.99
Quinine parenteral	3 (0.3 – 49)	17.6 ± 4.31	24.2 ± 4.96	36.6 ± 5.7
Quinine parenteral + Artesunate ir	45 (32 – 60)	-	-	-
Artesunate ir, single – 20 mg/kg	6.3 (2 – 30)	19.4 ± 1.63	19.4 ± 1.63	20.7 ± 0.99

* ir: Intra-rectal

### Efficacy

#### 1 comparisons with quinine

**Figure 1:**
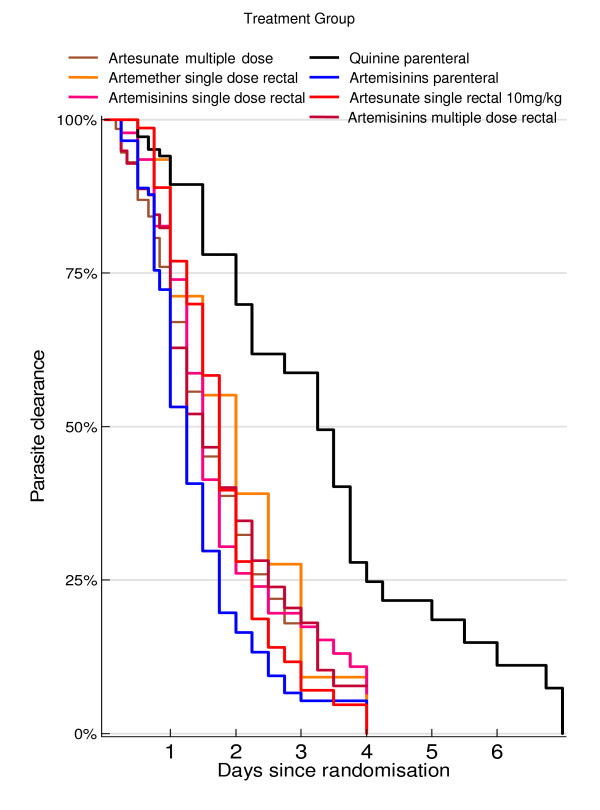
Cumulative probability of having parasites: parenteral quinine versus treatment with artemisinins.

Two studies (Molyneux1997–8 and Barnes1998) contributed to a standard meta analysis as both studies compared clinical and parasitological response of artesunate 10 mg/kg versus quinine 10 mg/kg. The log-transformed PRR at 24 h with a single dose of rectal artesunate was significantly better than quinine, weighted mean difference 0.60 (95% CI 0.32–0.89, p ≤ 0.0001). The pooled analyses showed that the artemisinin derivatives, regardless of route of administration and number of doses, were superior to quinine in reducing parasitaemia at 12 and 24 hours (Figure 1 and Table 3). In the model, parasitological efficacy was partly dependent on age and severity of disease but independent of baseline parasitaemia and region of use.

**Table 3:** Parasite reduction ratio at 12 and 24 hours compared with quinine: mixed model results

	**12 hr final model**			24 hr descriptive model**			**24 hr final model**	
	**%**	**p-value**	**N**	**%**	**p-value**	**N**	**Odds Ratio**	**p-value**
**Single dose Rectal Artemisinin vs Parenteral Quinine**								
Parenteral quinine	48.6		35	68.7		30		
Rectal artemisinin	72.3	0.337	46	95.5	0.29	46	8.2*	0.000
**Multiple dose Rectal Artesunate vs Parenteral Quinine**								
Parenteral Quinine	27.5		123	67.1		106		
Multiple rectal artesunate	56.5	0.04	272	89	0.004	254	11.03	0.009
**Multiple Rectal Artemisinin vs Parenteral Quinine**								
Parenteral Quinine	48.6		35	68.7		30		
Multiple rectal artemisinin	63.3	0.343	98	89.1	0.090	80	3.70*	0.004
**Single dose Rectal Artesunate vs Parenteral Quinine**								
Parenteral	36		293	63.7		106		
Rectal	79	0.000	123	99	0.000	264	23.5	0.000
Age 5–14 yrs***	-12	0.043		-9.4	0.015		0.33	0.015
Age >14 yrs***	-18	0.026		-6.9	0.152		0.32	0.021
Severe disease				11.5	0.045			

* No solution was found for the mixed model. Corresponding non-hierarchical regression used instead.

** Descriptive estimates of parasite reductions at 24 hours are provided only for comparisons with 12 hour estimates, see section 'Statistical analysis'

*** Reference category is <5 years

Time to clearance of parasitaemia for the different drugs are given in Figure 1. Multiple Cox regression analysis showed a significant difference in time to clearance between parenteral artemisinins and parenteral quinine (Hazard Ratio HR = 4.1; p ≤ 0.0001), between single dose rectal artesunate and parenteral quinine (HR = 2.7; p ≤ 0.0001) and between single dose artemisinin suppositories and quinine (HR = 2.4;p = 0.03) with parasitaemia at baseline being a significant covariate in the parenteral comparison (HR = 0.99; p ≤ 0.0001) and in the comparison with single dose artesunate (HR = 0.99; p = 0.042), but not in the comparison with single dose artemisinin suppository treatment.

#### 2 Comparisons between artemisinin derivatives

Mixed model estimates comparing efficacy of the different artemisinin derivatives are provided in Table 4.

**Table 4:** Parasite reduction ratio at 12 and 24 hours: Mixed model results

	**12 hr final model**			24 hour descriptive model**			**24 hr final model**		
	**%**	**p-value**	**N**	**%**	**p-value**	**N**	**Odds Ratio**	**p-value**	**N**
**Single Rectal Artemisinin or Artesunate vs Parenteral Artemisinins**									
Parenteral	45.7		225	83.8		49			179
Rectal	59.1	0.018	339	90.0	0.069	331	1.75*	0.072	201
Severe malaria	16.6	0.008							
**Multiple Artesunate vs Single Artesunate**									
Single artesunate	57.7		169	93.7		80			155
Multiple artesunate	32.7	0.001	249	87.5	0.279	307	0.19	0.004	232
Total dose at 24 hours	19.5	0.011					1.14	0.008	
**Single Artemisinin vs Single Artesunate**									
Single artesunate	64.2*		169	93.9		46			46
Single artemisinin	72.3	0.262	46	95.5	0.792	155	1.13*	0.813	155
**Multiple Artemisinin vs Single Artemisinin**									
Single artemisinin	72.3		46	95.5		80			80
Multiple artemisinin	63.2	0.545	98	89.1	0.348	46	0.60*	0.403	46
**Single Artemether vs Single Artesunate**									
Single artesunate	73.5		51	96.7		109			109
Single artemether	53.5	0.000	124	83.1	0.000	41	0.22*	0.002	41

* No solution was found for the mixed model. Corresponding non-hierarchical regression used instead.

** Descriptive estimates of parasite reductions at 24 hours are provided only for comparisons with 12 hour estimates, see section 'Statistical analysis'

##### 2.1 Parenteral versus rectal administration

##### Parenteral artesunate or artemether versus single-dose artemisinin or artesunate rectal administration

**Figure 2:**
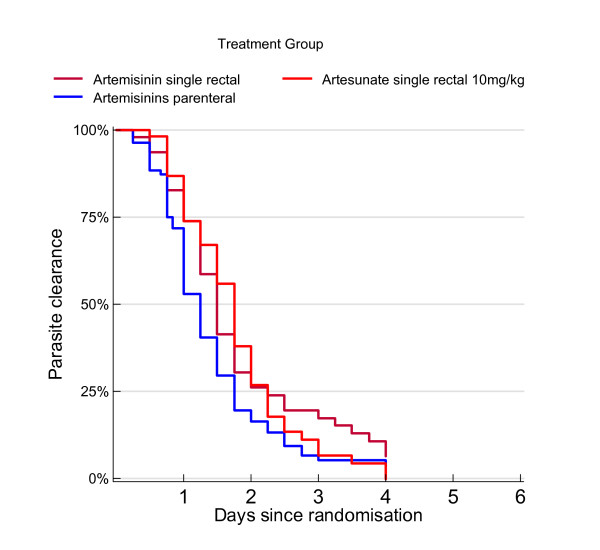
Cumulative probability of having parasites: parenteral artemisinins versus rectal artemisinins.

Mean PRR using either a single dose artesunate or single dose artemisinin was higher than parenteral artemisinins at 12 hours (65.9% for rectal treatment compared with 60.0% for parenteral treatment). This was observed also in the adjusted model presented in Table 4, in which severe disease was an important covariate in response prior to 12 hours. At 24 hours the difference was in the same direction, with 90.0% reduction in parasitaemia with rectal artemisinins compared with 83.8% parasite reduction with parenteral artemisinins. The proportion PRR>90 at 24 hours gave an OR = 1.75 (p = 0.072) in favour of rectal administration. However, the Kaplan Meier survival plot provided in Figure 2 demonstrates the overall superiority of parenteral administration beyond the 24-hour period (unadjusted log rank test of survival function p ≤ 0.0001 for a single dose of rectal artesunate/artemisinin versus parenteral treatment). Baseline parasite count was a significant covariate (p ≤ 0.0001) in the Cox regression giving an adjusted HR = 0.52 (p ≤ 0.0001) for a single rectal treatment versus parenteral treatment.

#### 2.2 Response between different derivatives given rectally

##### Multiple dose artesunate versus single dose artesunate

All multiple dose artesunate studies were undertaken in Asia and regression analyses were limited to patients from this region. The crude PRR at 12 hours with a multiple dose was 52.6% compared to a single dose of rectal artesunate at 64.1%. In the adjusted model (Table 4), the PRR at 12 hours was 32.7% with multiple dose treatment and 57.7% with single dose therapy, with severity of disease a significant covariate (OR = 19.5, p = 0.011).

At 24 hours, the mean PRR was 87.5% for the multiple dose treatment and 93.7% for the single dose. An adjusted logistic regression model indicated that a patient with moderately severe or severe disease had a five times greater chance of achieving a 90% reduction of parasitaemia at 24 hours with single dose artesunate rectal treatment (multiple dose compared with single OR = 0.19 (p ≤ 0.0001)), and that the total dose over 24 hours was the only variable independently influencing this outcome (Table 4). In all studies with multiple dose rectal administration with artesunate (all in Asia), a 200 mg suppository was provided sequentially at intervals over 72 hours, resulting in a mean exposure of 7.9 mg/kg over 12 hours and 14.9 mg/kg over 24 hours. Single dose studies attempted to provide a dose as close to 10 mg/kg as possible at initiation of therapy; the mean dose was 8.5 mg/kg over 24 hours (10.1 mg/kg in studies in Thailand).

The Kaplan-Meier analysis (log-rank test p = 0.45) and Cox regression adjusted analysis indicated no significant difference in the time to clearance of parasitaemia between multiple and single dose rectal artesunate treatment (HR = 1.04 p = 0.76), the assumption of proportional hazards not being justified. However, parasitaemia at baseline(HR = 0.99), total treatment dose prior to 12 hours (HR = 1.1), and patient age (HR = 0.64) were three significant covariates in this analysis (all at p = 0.0001).

##### Single dose artemisinin versus single dose artesunate

Two Vietnamese studies with single dose artemisinin given rectally over 24 hours were compared with single dose artesunate studies in Asia. Mean PRRs at 12 hours with single dose artemisinin and single dose artesunate suppositories were 72.3% and 64.2%, respectively. Corresponding PRRs at 24 hours were 95.5% and 93.9%, respectively, with OR = 1.13 (p = 0.813). The Kaplan Meier analysis (log-rank test p = 0.71) and Cox regression analysis did not show a significant difference in time to parasite clearance between the two treatments (HR = 1.07, p = 0.73). The only covariate of significance was parasite count at initiation of treatment (HR = 0.99, p = 0.023).

##### Multiple dose artemisinin versus single dose artemisinin

There were three studies in the comparison: one study with single dose artemisinin over 24 hours and two studies with multiple dose artemisinin, all conducted in Vietnam. Mean PRR at 12 hours was 72.3% with single dose artemisinin suppositories and 63.2% for multiple dose artemisinin. No covariate, including total dose which was significantly higher in the multiple dose group (20 mg/kg versus 45 mg/kg, p ≤ 0.0001), influenced outcome in the adjusted model at 12 hours. At 24 hours, the crude mean values of PRR were 89.1% for multiple dose artemisinin treatment and 95.5% for single dose treatment with an OR = 0.60 p = 0.40. The Kaplan Meier survival analysis (log rank test p = 0.98) and the adjusted Cox regression analysis of multiple versus single (HR = 1.24, p = 0.27) did not indicate any difference in time to parasite clearance between the two groups although parasite count at baseline was a significant covariate (HR = 0.99, p = 0.05). Patient weight data was not available to enable estimation of the effect of dose (mg/kg).

##### Single dose artemether versus single dose artesunate

Only one study with artemether suppositories was performed (Uganda), with a single rectal dose of artemether being given once daily for 7 days. Mean PRR at 12 hours was 53.5% for single dose rectal artemether compared with 73.5% with single dose artesunate (p ≤ 0.0001). No covariate influenced this outcome significantly. At 24 hours, mean PRR was 83.1% for single dose artemether and 96.7% with single dose artesunate (p ≤ 0.0001). The odds of achieving a 90% reduction in parasitaemia at 24 hours was about one fifth for artemether compared to artesunate (OR = 0.22, p = 0.002) in a moderately severe or severe patient. Mean total treatment dose during the initial 24 hours with single dose artemether was 6.72 mg/kg compared with 9.4 mg/kg with single dose artesunate. The Kaplan Meier analysis (log-rank test p = 0.18) and Cox regression analysis (HR = 1.56, p = 0.21) of time-to-clearance of parasitaemia showed no significant differences between the two types of artemisinin-based suppositories: artemether given once daily for 7 days versus artesunate given only once in the first 24 hours and followed by sulphadoxine-pyrimethamine (HR = 1.56, p = 0.21).

##### Effect of consolidation treatment on recrudescence

**Figure 3:**
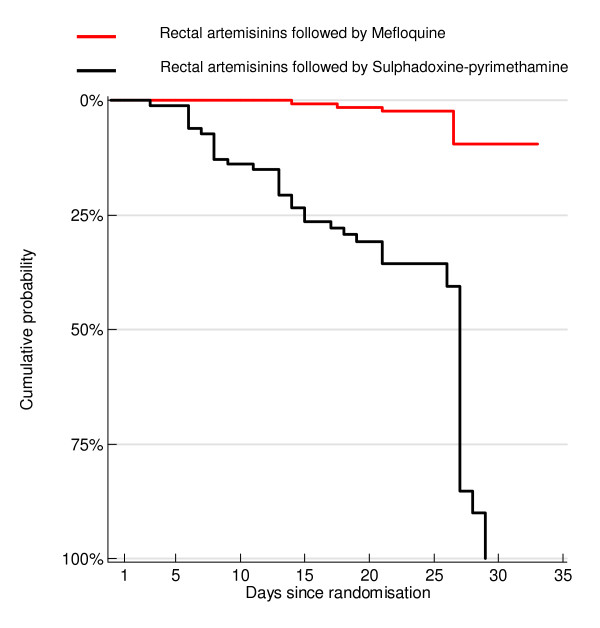
Cumulative probability of recrudescing with consolidation treatment.

Consolidation treatment varied (Table 5). A multiple Cox regression analysis of time-to-recrudescence gave a non-significant difference between an intra-rectal and parenteral artemisinin derivative when both were followed by mefloquine (HR = 0.78, p = 0.639) but parasitaemia at 72 hours was a significant covariate in the analysis (HR = 0.99, p = 0.003). However, in a comparison of intra-rectal treatment followed by sulphadoxine-pyrimethamine (SP) versus intra-rectal treatment followed by mefloquine, the Kaplan Meier analysis (log-rank test p ≤ 0.0001) and Cox regression analysis (HR = 0.36, p = 0.006) of time to recrudescence between the two treatments showed a significant difference in favour of mefloquine, with parasitaemia at 72 hours (HR = 0.99, p = 0.005) and region of study (HR = 0.16, p = 0.001) being significant covariates in the analysis (Figure 3).

**Table 5:** Consolidation treatment used in different clinical studies

**Initial Treatment group**	**Consolidation Treatment**	**Follow-up period**	**Study Identification/Year of Enrolment (Ref)**	**Total patients**
Artemether ir*, single dose	Artemether ir	7 days	Aceng 2002–3 [32]	51
Artemisinin ir, single dose	Mefloquine + SP	72 hours	Hien 1998	46
Artemisinin ir, multiple dose	Mefloquine	None post discharge	Phuong 1992–5 [31]	46
			Vinh 1992–4 [33]	52
Artesunate ir, multiple dose	Artesunate + SP	72 hours	Karunajeewa 2003–4 [34]	41
	Mefloquine	28 days	Looareesuwan 1995 [35]	60
			Than 1998	100
		None post discharge	Bhatt 1994–5 [36]	23
	Chloroquine or SP	None post discharge	Karunajeewa 2001 [37]	48
Artesunate ir, single dose	Artesunate + Mefloquine	28–42 days	Van Vugt 1997–9	44
		28 days	Looareesuwan 2000	69
	Mefloquine	28 days	Looareesuwan 1996	26
	Chloroquine or SP	30 days	Krishna 1996 [10]	23
	SP	28–42 days	Molyneux 1997–8 [30]	113
	Mefloquine-SP	72 hours	Hien 1998	44
Artesunate per os	Artesunate + Mefloquine	28–42 days	Van Vugt 1997–9	17
Artemisinins parenteral	Artesunate + SP	72 hours	Karunajeewa 2003–4 [34]	38
	Mefloquine	28 days	Looareesuwan 1996	24
		None post discharge	Phuong 1992–5 [31]	40
			Vinh 1992–4 [33]	123
	Chloroquine or SP	30 days	Krishna 1996 [10]	11
Quinine parenteral	Quinine	7 days	Aceng 2002–3 [32]	52
	SP	28–42 days	Barnes 1998 [30]	36
		None post discharge	Phuong 1992–5 [31]	35
Quinine parenteral + Artesunate ir	SP	42 days	Barnes 1998 [30]	5
Grand Total				1167

* ir: Intra-rectal

##### Safety

A total of 196 adverse events were reported in 140 (17.8%) of the 786 patients treated with rectal artemisinins (Table 6). By comparison 67 adverse events were reported in 30 (24.3%) of 123 patients treated with parenteral quinine.

**Table 6:** Adverse events noted in patients treated with suppositories and parenteral treatment, by treatment group.

	**Rectal artemisinin**	**Non-rectal artemisinin comparator**	**Non-artemisinin comparator (quinine)**	**TOTAL**
Total no. patients included in analysis	786	253	123	1162
Total no. (%) of patients in whom one or more adverse event reported	140 (18)	24 (9)	30 (24)	194
Total no. of adverse events:	196	43	67	306
Classification (aetiology):				
Possibly drug-related	37	14	27	78
Not likely to be drug related	105	28	40	173
Unable to be classified	54	1	0	55
Classification of possibly drug-related events according to body system:				
Generalised	11	1	0	12
Neurological	1	1	7	9
Digestive	18	10	8	36
Urogenital	1	1	0	2
Haemopoetic	3	1	4	8
Special senses (hearing)	3	0	5	8
Other	0	0	3	3

Of the 196 adverse events in patients treated with suppositories, 37 events in 21 patients were considered to be potentially drug-related, based on classifications provided by the clinical investigators. A further 105 events in 69 patients were classified as non-drug related and 50 events in 54 patients could not be or were not assigned a cause. Therefore, overall, 2.7% (21/786) of all rectal artemisinin-treated patients were thought to have had a potentially drug-related adverse event, 8.8%(69/786) a non-drug related adverse event and an additional 6.4% (54/786) had an adverse event of uncertain causality. By comparison, 27 of 67 (40.3%) reported adverse events occurring in 123 quinine-treated patients were considered drug-related meaning that 22.0% (27/123) of quinine-treated patients experienced an adverse event that was considered potentially drug-related.

Approximately 29.7% (11/37) of suspected drug-related adverse events in rectal artemisinin-treated patients were defined as affecting the body as a whole (including fever, headache and unspecified pain), 2.7% (1/37) were related to the nervous system (dizziness), 8.1% (3/37) were related to the special senses (hearing impairment) and 48.6% (18/37) related to the gastrointestinal system (vomiting, nausea, diarrhoea, constipation, abdominal pain). For those in the quinine comparator group, 25.9% (7/27) of adverse events were related to the nervous system, 29.6% (8/27) to the digestive system, 18.5%(5/27) affected special senses/hearing and 14.8% (4/27) the haemopoetic system. A meaningful comparison of safety profiles between the different artemisinin products was beyond the scope of this analysis. It should be noted that most of the safety data presented here are from patients treated with either artesunate (591) or artemisinin suppositories (144).

In summary, the total incidence of adverse events considered by clinicians to be possibly drug-related was estimated at being between 2.7% and 9.0% of all rectal artemisinin-treated patients, compared with 22% of quinine-treated patients. The majority of possibly drug-related adverse events in rectal artemisinin-treated patients involved either the gastrointestinal system or were generalized and non-specific in nature and were not severe.

## Discussion

This review addresses the lack of any data directly comparing the therapeutic efficacy and safety of the different rectal preparations of artemisinin derivatives. The pooled analysis of individual patient data suggest that artemisinin and artesunate suppositories rapidly eliminate parasites and are safe. There is far less evidence for artemether [32] and no studies of dihydroartemisinin suppositories were available to be included in this analysis. The results indicate that both artemisinin and artesunate, whether as single or multiple dose regimens, induce a superior parasitological response than parenteral quinine over the 24 hours following initiation of treatment. Regimens employing a higher single dose of rectal artesunate were five times as likely to result in >90% parasite reductions at 24 hours than were multiple lower doses of rectal artesunate or than a single administration of artemether. These results imply that dosage regimens that result in immediate high blood concentrations of drug [10,30,34,38] are those best able to reduce parasitaemia in patients with evolving severe malaria and that sustained drug exposure achieved by sequential treatment with moderate doses [20,21,35,39] offers no therapeutic advantage.

The analysis used the rate of parasite clearance in the first 24 hours following treatment as the primary endpoint to compare therapeutic efficacy of alternative drugs and regimens. This endpoint has been commonly used in studies of antimalarial efficacy, particularly for treatments intended for severe malaria [40]. Parasite clearance is a surrogate marker of clinical response but it cannot be assumed that superior parasite clearance equates with improved clinical outcome and lower mortality. Although parenteral artemisinin derivatives have long been recognised as having superior parasite clearance to quinine, it remained uncertain until recently whether this characteristic converted into a survival benefit. A recent trial comparing iv artesunate with parenteral quinine demonstrated a 30% lower mortality with artesunate, confirming that more rapid initial parasite clearance may translate to reduced mortality in severe adult malaria [41]. Therefore, although the current pooled analysis was not powered to assess mortality as an endpoint, the differences in parasite clearance rates between rectal artemisinins and parenteral quinine, and between different rectal artemisinin dosing regimens, should be regarded as important indicators of possible real differences in therapeutic efficacy and clinical benefit. It should also be noted that any future study designed to use mortality as an endpoint to compare different rectal artemisinins (or to demonstrate non-inferiority of a rectal artemisinin with a parenteral preparation such as iv artesunate) would require such a large sample size that it is unlikely ever to be implemented. Therefore the surrogate marker of parasite clearance used in this analysis is likely to remain the best available evidence on which to base comparisons of treatment efficacy for the rectal artemisinins.

There has been a recent systematic review of published data on rectal artemisinin derivatives with a focus on pharmacokinetics of various preparations and a summary of efficacy[42]; however there were less data and limited capacity to standardize definitions and account for statistical heterogeneity. In contrast, the current meta-analysis synthesizes individual patient data from studies meeting well-defined inclusion criteria and for whom standardized end-points were calculated. The analysis has enabled a robust and statistically powerful comparison of efficacy outcomes between rectal artemisinins, parenterally administered artemisinins and parenteral quinine that has had the capacity to examine and allow for the influence of covariates such as age, geographic origin and disease severity. Given that only a small number of direct comparative trials have been performed, this meta-analysis of 1162 individual patients represents a significant contribution to the available comparative efficacy data on rectal artemisinins. In particular, its results showing that early parasite clearance of rectal artemisinins is clearly superior to that of quinine, and appears equivalent to that of parenteral artemisinins is an important observation, given the results of the recent SEQUAMAT trial[41].

There are methodological limitations inherent in making comparisons of safety across several trials and in attributing causality to adverse events in patients with malaria. Overall the data suggest that the artemisinin based suppositories studied have a benign safety profile, consistent with that of the artemisinins in general[43,44]. There were no special concerns related specifically to the rectal route of administration and there were no reports suggestive of serious neurotoxicity. Neurotoxicity of the artemisinin derivatives has been described in animals but this now appears to be associated with sustained exposure to the central nervous system rather than peak levels [45]. Therefore a single dose as pre-referral treatment (rather than multiple dosing) may also have additional theoretical benefits in terms of safety as well as efficacy. Pharmacokinetic data have largely been derived from studies of artesunate[10,20,21,30,31,38,47] and artemisinin[14-18,48,49] with little data on rectal formulations of artemether[32] or dihydroartemisinin[22]. In the absence of sufficient pharmacokinetic information it cannot be assumed that all rectal preparations have the same efficacy or safety profile. Well-designed clinical trials that directly compare the efficacy, safety and pharmacokinetic profile of the different suppository formulations are needed.

The evidence from this analysis supports the WHO recommendation for the use of artesunate and artemisinin as initial pre-referral treatment[8]. The analysis was not designed to assess long-term cure rates and there are insufficient data on which to substantiate the use of rectal treatment for full management of severe malaria.

## Conclusion

Early effective treatment with artemisinin based suppositories has potential as a lifesaving intervention, particularly at the periphery of the health-care system, where suppositories might be administered early in lieu of parenteral treatment in remote communities by relatively untrained personnel. Combined with accurate diagnosis and artemisinin combination therapy, rectal artemisinins have been effectively used to reduce malaria incidence and mortality in Asia [3,50,51], an approach which holds great promise for malaria control elsewhere.

## Competing interests

Dr Karunajeewa has received funding for investigator-driven research from Mepha pharmaceuticals, Aesch-Basel, Switzerland, manufacturers of artesunate suppositories and has received honoraria for writing technical reports for the same company. No other authors have a conflict of interest.

## Authors' contributions

The authors accept full responsibility for the overall content of this report. MG and MW are staff members of the World Health Organization. IR and MW established and executed the search strategy. MG contacted the authors of published and unpublished material for individual patient data; IR and MP extracted the efficacy data and MW and MG extracted and analysed the safety data with HK. MP led the statistical analysis of efficacy conducted and interpreted by IR and MG. MW, HK and MG conducted the safety analysis. All authors reviewed and interpreted the results and analyses. MG wrote the first draft, reviewed and finalized by all authors. All authors read and approved the final manuscript. The authors alone are responsible for the views expressed in this publication and they do not necessarily represent the decisions, policy or views of the World Health Organization.

## Pre-publication history

The pre-publication history for this paper can be accessed here:


